# VertNet: A New Model for Biodiversity Data Sharing

**DOI:** 10.1371/journal.pbio.1000309

**Published:** 2010-02-16

**Authors:** Heather Constable, Robert Guralnick, John Wieczorek, Carol Spencer, A. Townsend Peterson, The VertNet Steering Committee

**Affiliations:** 1 Museum of Vertebrate Zoology, University of California Berkeley, Berkeley, California, United Stated of America; 2 Department of Ecology and Evolutionary Biology, University of Colorado, Boulder, Colorado, United States of America; 3 Biodiversity Research Center, University of Kansas, Lawrence, Kansas, United States of America

## Abstract

Responding to the urgent need to make biodiversity records broadly accessible, the natural history community turned to “the cloud.”

Biodiversity is in a crisis caused by multiple human impacts on the environment [1]–[3]. The immediate and critical tasks in addressing this crisis are to examine global biodiversity patterns and document changes through time and space in order to understand the factors contributing to loss of biodiversity [4]. Meeting this challenge has emerged as a global priority [5]–[7] that requires approaches to mobilize data across broad geographic and taxonomic ranges.

The community of vertebrate natural history collections has begun to meet this challenge by establishing social and technological infrastructures that provide open access to species occurrence data through broad participation and funding from the US National Science Foundation (NSF) and the Global Biodiversity Information Facility (GBIF). One result is VertNet, a publicly accessible database of vertebrate biodiversity data from natural history collections around the world. VertNet currently consists of four existing global vertebrate networks: Mammal Networked Information System (MaNIS) (http://manisnet.org – mammals [8]); Ornithological Information System (ORNIS) (http://ornisnet.org – birds); HerpNET (http://herpnet.org – amphibians and reptiles); and FishNet 2 (http://www.fishnet2.net – fishes). These networks collectively mobilize over 52 million records from over 70 institutions, which represent about 70% of all the vertebrate species occurrence data that are accessible through GBIF. VertNet was created to develop the tools and infrastructure necessary to make the data in these distributed networks available in a standard format to maximize their potential for understanding and protecting biodiversity. GBIF and VertNet work synergistically to enhance biodiversity data mobilization efforts. GBIF has identified the important role that VertNet will play in its new emphasis on decentralization of services and applications [5]. In particular, VertNet provides important data maintenance services, including data cleaning and indexing, thus removing development and deployment burdens for many fundamental tasks from GBIF.

Data from VertNet are currently accessed through the four networks by a broad audience at a high frequency of about 2.5 million records per week. The networks continue to grow, even those whose extramural funding have expired; MaNIS, for example, has grown from 17 contributing institutions under the original grant to 38 institutions, with a waiting list of 31 more that will be added to the network as time and resources permit. The same enthusiasm is mirrored across the other three vertebrate networks. This rapid growth demonstrates an important sociological shift from skepticism to enthusiasm for data sharing.

Ironically, the success of these networks has become their biggest challenge, straining original architectures and demanding a scalable and more sustainable solution. Below, we provide perspectives on the sociological and technical developments that brought vertebrate biodiversity networks to this point and discuss solutions to the immediate and anticipated challenges.

## Developing Data-Sharing Technology

The fundamental concept underlying the vertebrate biodiversity networks is that data contributors are the primary and authoritative source for information about the occurrence data over which they have custody. The networks merely facilitate access and sharing of these distributed primary resources. A fully decentralized architecture, with all requests distributed directly to the primary sources, highlighted the primacy of the contributing institutions and was an essential phase in promoting participation, instilling confidence and a sense of control within the community.

The current system relies on a data standard [9] and a distributed query protocol [10]. The Darwin Core specifies terms—such as scientific name, date, and locality descriptions—provide information about species' occurrences in nature. Distributed generic information retrieval (DiGIR) specifies the messaging system that allows questions to be asked and answered across the network of primary data sources. The networks grow by having prospective contributors establish access to their Darwin Core–compliant data through networked computers outfitted with DiGIR and a Web server, and then by requesting that these servers be registered to participate in one or more of the networks. Successful installation and registration makes the new resources available, allowing them to be accessed simultaneously with those of their contributing peers so that each network simulates access to a single data store of all of the contributors' data.

## Collaboration, Training, and Data Improvement

One of the strongest incentives for organizations to participate in the vertebrate biodiversity networks has been the promise of improved data quality, focusing particularly on converting textual locality descriptions into spatial formats for mapping [11],[12]. Through remote collaborations, 64 institutions to date have shared the enormous task of georeferencing the network contents. Collectively, nearly 4.5 million occurrence records from 867,000 distinct locations have been georeferenced following best practices [13] by leveraging geographic resources and expertise at each institution. Since 2003, at least 175 undergraduates and 282 higher level researchers from 161 institutions in 40 countries have been trained directly through project activities, including 14 international georeferencing workshops. In addition to increasing technical understanding and capacity among contributors, these collaborations have produced effective economies of scale and provided a vibrant exchange of expertise among participants. Whereas georeferencing was an intentional deliverable of grants supporting the vertebrate networks and an obvious benefit for contributors, participation has also revealed unforeseen benefits to the primary data custodians through data quality improvement from user feedback. It has become clear that the vastly increased exposure and use of the data have revealed and motivated correction of erroneous information that otherwise may have gone undetected.

## New Challenges

The current networks were designed to rely on live connections to contributors' server installations to achieve two goals: (1) to support contributor confidence that their participation was under their own control; and (2) to emphasize the primacy of the original source. The unforeseen growth of the networks has revealed performance bottlenecks, as well as other scalability and sustainability problems. Because every request for data is propagated to the networked sources, aggregate responses are limited by the slowest responder, and data from contributors not connected at the moment of the query are simply unavailable until they come back online. These limitations make it impractical to provide users with dynamic information about network content in advance of a specific search. Users have no way of knowing the expected content of individual fields or of overall content of the network, such as which countries or taxa are represented, or how many total records are available. Network responsiveness is clearly at the mercy of each of its distributed components.

The potential of the networks to improve the quality of their holdings is also hampered by the current architecture. Although data sets comply with the agreed-upon schema (Darwin Core), data content is inconsistent among contributors and has limited quality-assessment information. These deficits could be overcome most effectively with collaborative tools and feedback mechanisms associated with the networks—tools such as collaborative georeferencing workbenches, vocabulary look-up services, and taxonomic authorities.

We have managed the current networks through concerted efforts from both network and participant personnel. The National Biological Information Infrastructure of the US Geological Survey has established a programmer position to provide network support to meet the growing demands on the systems. In the first nine months following the inception of this position in 2008, the programmer provided support to 73% of the contributing installations. The estimated total annual operating cost (people and hardware) across the four vertebrate networks at the 2009 participation level was $195,600.

## VertNet as a New Model for Biodiversity Networks

VertNet proposes a new model for biodiversity networks in which the computing resources are consolidated on “the cloud” utilizing the Google App Engine platform as a service. Cloud computing is typically a pay-per-use model utilizing an Internet-based third party, a dynamically scalable and often virtualized computing resource. Such a model removes the requirement and cost to contributors to buy or maintain their own servers while leveraging all the data integrity and replication services provided by the cloud. Under the new model, contributors would use a Web-based administrative interface to create a “provider” in the cloud. The process would allow contributors to describe (i.e., provide metadata for) their data sets, define usage restrictions and citation information, add contact information, and later access information about usage statistics. After creating their provider, contributors would download and use a local application to publish network-ready data—data conforming to the Darwin Core—to the cloud. Subsequent updates would use the same local application to publish only differences (additions, changes, deletions) since the previous publishing act. The data store in the cloud will contain the primary data published from all contributors as persistently available records, uniquely identifiable by their data store key. The data store will also contain summary information about the aggregate of all data and associated data from other sources, such as auxiliary data look-ups, user feedback, and data quality assessments. All of these would be accessible and downloadable from the data store through an Application Programming Interface (API) and through Representational State Transfer (REST).

**Figure 1:**
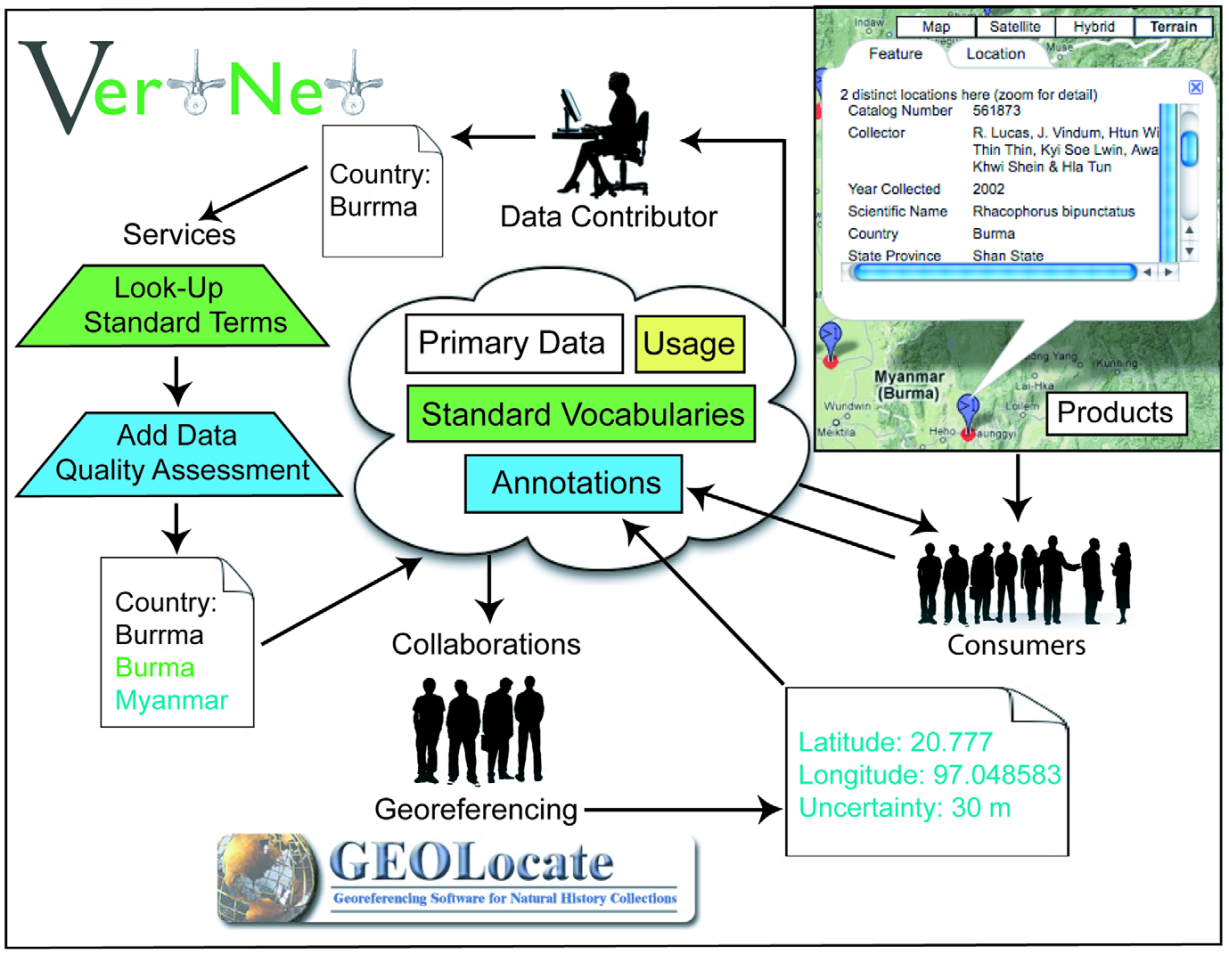
Interactions with the data store. The proposed cloud-based architecture will allow contributors to publish primary data to the cloud, augmented with equivalent standard vocabulary values (in green), and with data quality assessments, which add annotations about potential errors or updates (in blue). Applications can also interact with the data store and contribute recommended improvements, which are then accessible to contributors and other users alike. For example, the collaborative georeferencing tool GeoLocate (http://www.museum.tulane.edu/geolocate/) could be used to add coordinates and uncertainty estimates to the data store as annotations. Data consumers can view data products, such as maps, and they can also make annotations to the data store. All information is accessible by consumers and data contributors, who have the option of updating the primary data with all added value features.

VertNet is a radical departure from the current model, in which Web portals query data from contributors' server installations on demand to one in which contributors publish to a cloud-based data store. Network performance and scalability issues will be alleviated under this model, while the “traditional” primacy of the sources will be maintained through contributor-mediated updates. Further, cloud-based annotation tools will enable users to flag suspect records so that collection-based curators can check and correct data at the source. Consolidation in the cloud will create a variety of improvement opportunities that are impractical under the current model. Contributor-mediated publishing introduces the feasibility of adding automated data quality improvement services to the publishing workflow. The publishing activity will make it possible for the first time to alert users when data of interest change or enter the network (new records in an area or place of interest). The consolidated data store will make it possible to determine the nature of the content of the network as a whole (vocabularies, record counts, net rate of change of information, uniqueness measures of given contributor's data in the context of the entire network). The combined data store also will facilitate collaboration by providing not only a platform on which to store the results of collaborations (georeferences, user-provided annotations) associated directly with the primary data they are meant to improve, but also a platform on which to build innovative applications (e.g., analysis, visualizations, workflows; Figure 1). The new model supports all of these benefits at an estimated 16-fold reduction in annual operating costs.

## Conclusion

The vertebrate networks represent a social and technological success story. In only eight years, a global community of eager contributors has managed to mobilize an impressive contribution of publicly accessible biodiversity data in a standard format. The success began by understanding the scope, sociological requirements, and technological constraints of the community we were trying to serve and was made manifest through hard work and contributions by a supportive community. By ensuring that data remain curated at the source, and by showing the importance of data sharing to promote data citation and usage, we have grown past our original technology implementation and are ready to move into a long-term production environment that departs from the original model. The new cloud-based architecture promises to be sustainable and scalable far into the future. We believe the development process here is not unique to biodiversity data, rather, past successes, current challenges, and new solutions may all provide useful lessons and approaches to other communities that are coalescing to share data.

## Abbreviations

API: application programming interface
DiGIR: distributed generic information retrieval
GBIF: Global Biodiversity Information Facility
IWGSC: Interagency Working Group on Scientific Collections
MaNIS: Mammal Networked Information System
NAS: National Academy of Sciences
NSF: National Science Foundation
ORNIS: Ornithological Information System
REST: Representational State Transfer
